# A randomized, open-label, multicenter study of switching to brolucizumab with or without a loading dose for patients with suboptimal anatomically controlled neovascular age-related macular degeneration—the FALCON study

**DOI:** 10.1007/s00417-022-05591-z

**Published:** 2022-02-21

**Authors:** F. G. Holz, Steffen Schmitz-Valckenberg, A. Wolf, H. Agostini, K. Lorenz, A. Pielen, N. Feltgen, R. Guthoff, C. Quiering, A. Clemens, K. Jaeger

**Affiliations:** 1 Department of Ophthalmology and GRADE Reading Center, University of Bonn, Bonn, Germany; 2 Department of Ophthalmology and Visual Sciences, John A. Moran Eye Center, University of Utah, 65 North Mario Capecchi Drive, Salt Lake City, UT 84312 USA; 3 Department of Ophthalmology, University of Ulm, Ulm, Germany; 4 Eye Center, Faculty of Medicine, University of Freiburg, Freiburg, Germany; 5 Department of Ophthalmology, University Medical Center, Johannes Gutenberg-University Mainz, Mainz, Germany; 6 University Eye Hospital, Medizinische Hochschule Hannover, Hannover, Germany; 7 Eye Center, Faculty of Medicine, University of Goettingen, Goettingen, Germany; 8 Eye Hospital, Faculty of Medicine, University of Duesseldorf, Duesseldorf, Germany; 9 Novartis Pharma GmbH, Nuernberg, Germany; 10 Novartis Pharma AG, Basel, Switzerland; 11 Department of Cardiology and Angiology I, Faculty of Medicine, Heart Center, University of Freiburg, Freiburg, Germany

**Keywords:** Neovascular age-related macular degeneration, Anti-VEGF, Brolucizumab, Switch

## Abstract

**Background:**

Treatment initiation with brolucizumab, a new potent anti-vascular endothelial growth factor (VEGF) agent, is typically performed with three monthly injections (loading dose) and has been well studied in treatment-naïve patients. However, no clinical data are available yet on whether or not anti-VEGF pretreated patients also benefit from a loading dose. In the clinical setting, different heterogeneous treatment patterns are used as no clinical trial has addressed this so far in a head-to-head comparison. Therefore, the *FALCON* study is investigating whether patients with unsatisfactory response to previous anti-VEGF treatments benefit from a loading dose at the switch to brolucizumab treatment.

**Methods:**

*FALCON* is a 52-week, two-arm, randomized, open-label, multicenter, multinational study in patients with residually active neovascular age-related macular degeneration (nAMD) who will be randomized 1:1 and started with brolucizumab 6 mg loading (three monthly loading doses) or brolucizumab 6 mg non-loading (one initial injection) and consecutive treatment every 12 weeks, respectively. The primary objective is to demonstrate non-inferiority of the non-loading vs. loading arm in mean change of best-corrected visual acuity (BCVA) from baseline to the mean value at week 40 to week 52. Secondary objectives include the assessment of anatomical outcomes, treatment intervals, safety and tolerability.

**Results:**

*FALCON* will be the first study to assess treatment initiation with an anti-VEGF agent in a switch situation with or without loading dose in patients with nAMD.

**Conclusions:**

The results will support the optimization of treatment of patients with previous unsatisfactory anti-VEGF response. Therefore, we expect to see an impact on current clinical practice which has been established for more than a decade.

**Trial registration:**

Clinicaltrials.gov: NCT04679935, date of registration—22-Dec 2020; EUDRACT number: 2019–004763-53, date of registration—03 Dec 2019.


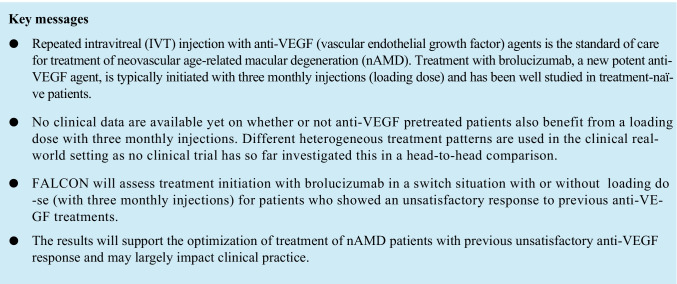


## Introduction

Age-related macular degeneration (AMD) is a leading cause of severe vision loss affecting approximately 8.7% of the worldwide population with projected numbers increasing up to 288 million people in 2040 [1].

The neovascular, exudative or wet form of AMD (nAMD) is characterized by retinal vascular leakage and fluid accumulation from neovascularizations. The use of intravitreal (IVT) injection pharmacotherapy targeting vascular endothelial growth factor (VEGF) has shown to improve visual outcomes in nAMD patients [2, 3]. Repeated IVT injection with anti-VEGF agents including the licensed ranibizumab and aflibercept, the non-licensed bevacizumab and the recently approved brolucizumab is used for treatment of nAMD [4, 5].

Brolucizumab is a humanized single-chain antibody fragment (scFv), binding to VEGF-A with high affinity. Its molecular weight of ~ 26 kilodalton allows a delivery of a high molar dose via IVT injection. A 6 mg dose of brolucizumab delivers a molar dose which is approximately 11 and 22 times higher than aflibercept 2 mg and ranibizumab 0.5 mg, respectively [6–8].

Brolucizumab was found to be efficacious and safe when compared to aflibercept in treatment-naïve nAMD patients in two large prospective randomized phase 3 trials (HAWK, HARRIER) [9, 10]. Treatment was started with three monthly brolucizumab injections (loading phase), followed by treatment every 12 weeks (q12w) or 8 weeks (q8w). The primary endpoint was met as brolucizumab demonstrated non-inferiority to aflibercept in mean change in best-corrected visual acuity (BCVA) from baseline to week 48 in both trials with more than 50% of patients being maintained on a q12w dosing interval. Brolucizumab also demonstrated superior anatomical outcomes versus aflibercept with fewer patients with intraretinal and/or subretinal fluid (IRF/SRF) and superior reductions in central subfield thickness (CSFT). The visual acuity gains and superior anatomic outcomes were maintained in the second year.

In terms of safety, HAWK and HARRIER described an incidence of intraocular inflammation (IOI) of 4% for brolucizumab 6 mg compared to 1% for aflibercept 2 mg-treated eyes. Most of these cases were reported as mild-to-moderate by the investigators, and the proportion of eyes that lost ≥ 15 letters was comparable between both groups at week 96. It is concluded that brolucizumab exhibited an overall well-tolerated safety profile [9, 10]. Based on the data of the phase 3 trials, brolucizumab has been approved for nAMD treatment in more than 40 countries. In the early post approval phase of brolucizumab use, retinal vasculitis (RV) and/or retinal vascular occlusions (RO), typically in the presence of IOI, were reported. The characterization of these adverse events was included in a safety label update by authorities. Management and treatment recommendations of these adverse events are given in expert consensus statements [11, 12].

Historically, anti-VEGF treatment is typically initiated with three monthly injections (loading dose), followed by a maintenance phase with either fixed (e.g. every 4 or 8 weeks) or individualized treatment intervals, based on treat-and-extend (T&E) or *pro re nata* (PRN) regimens [13]. Brolucizumab as the latest approved anti-VEGF provides the option to perform a q12w application directly after the loading dose [9, 10]. If, during the course of treatment, an anti-VEGF agent is found to be neither morphologically nor functionally effective without falling below its effective dose, a switch to another anti-VEGF agent may be appropriate. Several studies showed that switching to another anti-VEGF agent might lead to improvement in anatomical parameters and even stabilization or improvement of visual acuity [14–17]. For these switch patients, treatment is often initiated with a loading dose of three monthly injections, similarly as in treatment-naïve patients. However, no clinical data are available on whether anti-VEGF pretreated nAMD patients also benefit from a loading dose compared with a T&E or PRN regimen following a single injection. Therefore, different heterogeneous treatment patterns are applied in clinical practice as no clinical trial has addressed this so far in a head-to-head comparison.

The *FALCON* trial will investigate how to optimally initiate the switch to brolucizumab treatment after an unsatisfactory anti-VEGF treatment response and will assess treatment initiation with brolucizumab with or without loading dose. The results will support the optimization of treatment of nAMD patients with previous unsatisfactory anti-VEGF response.

## Materials and methods

### Study design

**Fig. 1:**
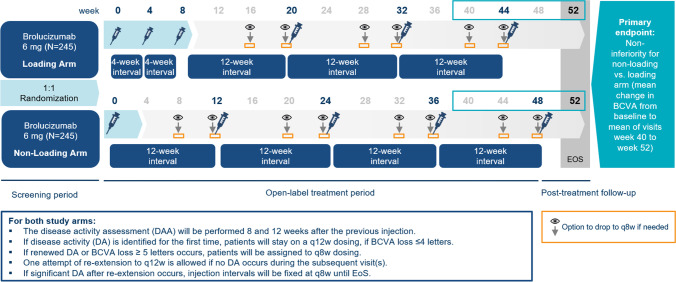
Study design of FALCON. BCVA best corrected visual acuity, EOS end of the study, q8w 8-week dosing interval

*FALCON* (Clinicaltrials.gov: NCT04679935; EUDRACT number: 2019–004767-53) is a 52-week, two-arm, randomized, open-label, multicenter, multinational study in patients with residually active nAMD. The study consists of three phases (Fig. 1). A screening period of up to 2 weeks will be used to assess eligibility (day − 14 to baseline). At baseline, eligible patients are randomized 1:1 and switched to either brolucizumab 6 mg loading (treatment initiation dose with three monthly initiation doses, referred to as loading in the manuscript) or brolucizumab 6 mg non-loading (one initial injection) and consecutive treatment every 12 weeks, respectively (open-label treatment period, baseline/day 1 to week 48). The last study assessment will be performed at week 52 (post-treatment follow-up period, week 48 to week 52).

During the open-label treatment period, the intended treatment schedule will be every 12 weeks with the option to adjust to an injection every 8 weeks depending on disease activity status. The disease activity decision should be based on the BCVA criterion (BCVA loss ≥ 5 Early Treatment Diabetic Retinopathy Study (ETDRS) letters or ≤ 4 ETDRS letters compared to the previous visit) and/or beyond that on the investigator’s judgment of visual and/or anatomic outcomes and signs of disease activity (e.g. IRF, SRF, hemorrhage, leakage, visual acuity loss over time etc.) The detailed criteria for the disease activity decision are listed in Fig. 1.

### Study population

The study population comprises male or female patients ≥ 50 years with active choroidal neovascularization (CNV) secondary to AMD, treated previously for this disease, who showed an unsatisfactory response to previous anti-VEGF treatments. The investigator will assess the eligibility of the patient and the study eye at the screening visit and confirm eligibility prior to randomization. Before enrollment, morphological eligibility will be confirmed by the central reading center as part of the screening period. If both eyes are eligible as per the inclusion and exclusion criteria described below, the eye with the worse visual acuity will be selected for study eye. Patients will be enrolled across two countries: Germany and Switzerland. The key inclusion and exclusion criteria are summarized in Table 1.

**Table 1:** Key inclusion and exclusion criteria

Inclusion criteria • Male or female patients ≥ 50 years of age at screening with signed written informed consent • Active CNV secondary to AMD that affects the central subfield, including retinal angiomatous proliferation (RAP) with a CNV component. If active CNV is not detectable in screening image data (no IRF and no SRF), presence of residual and/or recurrent fluid (IRF and/or SRF) within the last 6 months before baseline visit is also considered eligible. In this case, historical images must be submitted for analysis by the central reading center • Pretreatment with any anti-VEGF drug for a maximum of 5 years (60 months). Patients should have shown functional and/or anatomical treatment response to the pretreatment(s), prior to participating in this study • The treatment initiation phase with the current anti-VEGF must have been completed for at least 6 months with continuous treatment in a ≥ q4w to ≤ q12w injection interval (± 2-day window, i.e. 26 to 86 days inclusive) before the baseline visit. At least 4 weeks (minimum 26 days) must have passed between the last anti-VEGF pretreatment and baseline • BCVA score between 83 and 38 letters, inclusive, using Early Treatment Diabetic Retinopathy Study (ETDRS) visual acuity testing charts at both screening and baseline visit (study eye)
Exclusion criteria • Concomitant conditions or ocular disorders in the study eye at screening or baseline which could, in the opinion of the investigator, prevent response to study treatment or may confound interpretation of study results, compromise visual acuity or require planned medical or surgical intervention during the 52-week study period, atrophy or fibrosis at the center of the fovea as confirmed by central reading center or structural damage of the fovea (study eye) • Treatment with anti-VEGF drugs for > 5 years in the study eye • Any active intraocular or periocular infection or active intraocular inflammation, at screening or baseline (study eye) • Uncontrolled glaucoma defined as intraocular pressure (IOP) > 25 mmHg on medication, or according to investigator’s judgment, at screening or baseline (study eye) • Presence of amblyopia, amaurosis or ocular disorders in the fellow eye with BCVA < 20/200 at screening (except when due to conditions whose surgery may improve visual acuity, e.g. cataract) • Ocular treatments: pretreatment with brolucizumab at any time in the study eye, previous treatment with investigational drugs in the last 6 months, intraocular or periocular steroids at any time, macular laser photocoagulation or photodynamic therapy at any time, peripheral laser photocoagulation within 3 months prior to baseline, vitreoretinal surgery at any time, intraocular surgery within 3 months prior to baseline, aphakia with the absence of posterior capsule (study eye) • Stroke or myocardial infarction during the 6-month period prior to baseline • Systemic anti-VEGF therapy during the 3-month period prior to baseline

### Study objectives

*FALCON* is designed to assess efficacy and safety in patients switched to brolucizumab due to an unsatisfactory response to previous anti-VEGF treatments who will be treated with versus without a loading dose of three monthly intravitreal injections of brolucizumab. The primary objective is to demonstrate non-inferiority of the non-loading vs. loading arm in terms of mean change in best corrected visual acuity (BCVA) from baseline to mean of visits at week 40 to week 52. Secondary objectives include the treatment interval prolongation compared to previous treatment, the functional and anatomical outcomes comparing the two brolucizumab groups and safety and tolerability of brolucizumab treatment.

### Data collection

Patients will be enrolled in the study upon signing an informed consent. The screening and baseline visit will be used to assess eligibility and collect baseline characteristics, such as demographic data, medical history and nAMD characteristics. The morphological eligibility will be confirmed by the central reading center. The follow-up visits will take place every 4 weeks, adding up to a total study period of maximum 54 weeks. All visits include an ophthalmic exam as well as intraocular pressure and BCVA measurement. Moreover, spectral domain optical coherence tomography (SD-OCT) images will be taken at every visit. Color fundus photography, fluorescein angiography and optional OCT angiography will be performed at screening and week 52 (end of study visit). All images will be graded independently by the central reading center (CRC) which will be blind to the identity of the treatment arm from the time of randomization until database lock. Patients who discontinue the study treatment will continue in the study with all the scheduled assessments (except administration of study treatment and adherence to prohibited medication list). Those who prematurely withdraw from the study are scheduled for an early discontinuation visit. All patient data will be entered into an electronic case report form (eCRF).

### Safety measures

A number of safety precautions are installed to prevent potential serious adverse events. The details on safety precautions and monitoring have been provided to the investigators in the clinical trial protocol. Here, we would like to mention the essentials: First, patients with any active intraocular or periocular infection or active intraocular inflammation will be excluded from study enrollment. Moreover, before every injection, the investigator has to perform a thorough ophthalmic examination including anterior biomicroscopy (slit lamp examination) and posterior segment (indirect fundus) examination; if any signs of intraocular inflammation are present, an injection must not be performed, and the investigators must verify that these conditions are not present in the study eye prior to every injection. In patients developing intraocular inflammation, including retinal vasculitis and/or retinal vascular occlusion, treatment with brolucizumab in the FALCON study should be discontinued, and the events should be promptly managed, and additional images (OCT, FLA and CFP) will be taken. In addition, the patient will be instructed to contact the site immediately for any changes in vision and any symptoms of inflammation. More details on management and treatment recommendation of these adverse events are also provided in expert consensus statements [11, 12].

### Outcome measures

The primary efficacy endpoint based on BCVA was chosen to evaluate the benefits of treatment in terms of functional outcome. In addition, anatomical parameters like IRF, SRF and reductions in CSFT will be important outcome measures of treatment efficacy. Detailed objectives of the study are outlined in Table 2.

**Table 2:** Outcome measures of the FALCON study

Primary outcomes measure • Mean change in best-corrected visual acuity [baseline, week 40 to week 52], visual acuity test
Secondary outcome measures • Mean treatment interval [− 24 weeks, baseline, week 52], treatment interval distribution • Rate of patients with prolonged interval compared to mean treatment interval prior to enrollment [− 24 weeks, baseline, week 52], treatment interval distribution • Proportion of patients maintained at a every 12-week interval [every 12 weeks up to week 52], treatment interval distribution • Distribution of patients at every 8-week/every 12-week intervals [baseline and every 8 or 12 weeks, up to week 52], treatment interval distribution • Mean change in best-corrected visual acuity [baseline, week 52], visual acuity test • Proportions of patients with best-corrected visual acuity improvements of ≥ 5, ≥ 10 and ≥ 15 letters [baseline, week 52], visual acuity test • Proportion of patients with best-corrected visual acuity ≥ 69 letters [at week 52], visual acuity test • Mean change in best-corrected visual acuity [baseline, week 16 to week 28], visual acuity test • Change in central subfield thickness [baseline, week 52], Spectral domain optical coherence tomography • Absence of intraretinal fluid, subretinal fluid and sub-retinal pigment epithelium fluid in the central subfield [up to week 52], Spectral domain optical coherence tomography • Presence of active choroidal neovascularization leakage [at week 52], fluorescein angiography • Incidence of ocular and non-ocular adverse events [up to week 52]

### Statistical analysis

To test the primary efficacy variable, non-inferiority in terms of mean change in BCVA from baseline to mean of visits at week 40 through week 52, a two-sided 95% confidence interval for the treatment difference will be derived from a mixed model for repeated measures (MMRM) with factors treatment arm, baseline BCVA and age. In order to demonstrate non-inferiority, the lower limit of the two-sided 95% confidence interval for the treatment difference (non-loading vs. loading) must be greater than − 4 letters representing the non-inferiority margin.

A sample size of 490 patients (245 patients in each treatment arm) is required to be recruited in the study, so as to have 90% power to demonstrate non-inferiority at a one-sided alpha level of 0.025, assuming equal efficacy and a common standard deviation of 13 letters and accounting for a drop-out rate of ca. 10%.

## Results

The *FALCON* trial will investigate how to optimally initiate a switch to brolucizumab treatment after an unsatisfactory anti-VEGF treatment response including recurrent or recalcitrant macular edema and assesses treatment initiation with an anti-VEGF in a switch situation with or without loading dose. *FALCON* is to our knowledge the first head-to-head comparison of two different switch regimens and will inform on essential clinical parameters in patients with nAMD and residual disease activity.

The study is planning to recruit patients across approximately 65 centers in Germany and Switzerland. Recruitment is planned to be completed by April 2023; the overall study is expected to be completed by May 2024.

## Discussion

Brolucizumab demonstrated non-inferiority to aflibercept in mean BCVA change from baseline to week 48 in treatment-naïve nAMD patients in two large prospective randomized phase 3 trials (HAWK, HARRIER) [9, 10]. In these studies, treatment with brolucizumab was initiated with three monthly injections (loading dose), which is also recommended on the various labels worldwide. If, during the course of treatment, an anti-VEGF agent is found to be neither morphologically nor functionally effective without falling below its effective dose, a switch to another anti-VEGF agent may be appropriate. Some case reports are described where patients were switched from another anti-VEGF to brolucizumab; however, no prospective controlled switch study was reported at time of writing. Moreover, there is no clinical data available on whether anti-VEGF pretreated nAMD patients also benefit from a loading dose, which applies to all currently approved anti-VEGFs. Therefore, different heterogeneous treatment patterns are applied when patients are switched from one anti-VEGF to another as no clinical trial has addressed this so far in a head-to-head comparison. It is known from several clinical studies, that the loading phase plays an important role in short, but also long-term clinical outcomes. Consequently, all historical and currently conducted pivotal trials investigating anti-VEGF treatments in treatment-naïve nAMD patients had a loading phase at trial initiation [18, 19]. A possible hypothesis is that disease activity of treatment-naïve nAMD patients would need to be treated intensively at the beginning to achieve a situation of disease control as quickly as possible. Therewith, further damage of retina cells would be prevented, and long-term visual acuity outcomes would be optimized. Furthermore, there is at least information from real-world data that missing the loading phase in nAMD patients might translate into worse long-term visual outcomes [20].

A recent consensus statement described the central role of fluid accumulation in different anatomical compartments as a biomarker for treatment or re-treatment with an anti-VEGF [21]. In previous clinical trials with shorter acting anti-VEGF agents, a number of patients still showed persistent fluid [22, 23]. In patients who still have persistent fluid as a sign of a residual disease activity, it is questionable whether the loading phase is needed in a switch situation to another anti-VEGF agent. It could be hypothesized that a sub-control situation regarding disease activity is present and just changing to a more potent anti-VEGF agent might be enough to achieve the same visual outcome in comparison to the more intensive treatment with a ‘new start’ with a loading phase. It would be essential to understand if there is a difference between treatment-naïve patients who receive brolucizumab loading doses per approved label and pre-treated nAMD patients after the initiation of brolucizumab (with or without loading doses), be it on visual acuity outcomes or disease control outcomes over time [22, 23].

To our knowledge, *FALCON* is the first study evaluating the impact of non-loading versus loading in a head-to-head comparison in patients who did show an unsatisfactory response to their previous anti-VEGF treatment. Combining the information on all collected disease parameters, the results will support the optimization of treatment of nAMD patients with an unsatisfactory anti-VEGF response. The study will provide some guidance as to whether a loading dose is required when switching. Moreover, it will show whether brolucizumab can reduce persistent fluid and provides optimized fluid and disease control after the switch. Therefore, we expect an impact on current clinical practice which has been established for more than a decade. As the study addresses a wide range of issues that clinicians face today, its results are awaited with interest. *FALCON* is taking the first step and paving the way for the investigation of real-world medical questions in a clinical trial setting to support treatment decisions in nAMD patients in routine clinical care.

## Data Availability

Not applicable.
